# PMSeeker: A Scheme Based on the Greedy Algorithm and the Exhaustive Algorithm to Screen Low-Redundancy Marker Sets for Large-Scale Parentage Assignment with Full Parental Genotyping

**DOI:** 10.3390/biology13020100

**Published:** 2024-02-05

**Authors:** Lei Xia, Mijuan Shi, Heng Li, Wanting Zhang, Yingyin Cheng, Xiao-Qin Xia

**Affiliations:** 1 State Key Laboratory of Freshwater Ecology and Biotechnology, Hubei Hongshan Laboratory, Key Laboratory of Aquaculture Disease Control, Ministry of Agriculture and Rural Affairs, The Innovation Academy of Seed Design, Institute of Hydrobiology, Chinese Academy of Sciences, Wuhan 430072, Chinacyy@ihb.ac.cn (Y.C.); 2 College of Advanced Agricultural Sciences, University of Chinese Academy of Sciences, Beijing 100049, China

**Keywords:** parentage allocation, mixed families, parentage marker set, fish, molecular marker

## Abstract

**Simple Summary:**

The challenges and expenses associated with accessing pedigree information pose a clear technical weakness that impedes the widespread adoption of selective breeding schemes in aquaculture. In large offspring populations, such as fish, using a small number of markers can substantially reduce the cost of parentage assignment. Theoretically, it is feasible to calculate the smallest parentage marker set from a given marker pool by exhaustively enumerating all possible marker combinations when full parental genotypes are known. However, the sheer number of markers can render the exhaustive method impractical. To address this, we have developed an online software that utilizes exhaustive and greedy algorithms to identify the smallest possible parentage marker set for closed populations. The tool was tested and proven to be effective in narrowing down the number of markers, thus providing technical support for large-scale parentage assignment applications.

**Abstract:**

Parentage assignment is a genetic test that utilizes genetic characteristics, such as molecular markers, to identify the parental relationships within populations, which, in commercial fish farming, are almost always large and where full information on potential parents is known. To accurately find the true parents, the genotypes of all loci in the parentage marker set (PMS) are required for each individual being tested. With the same accuracy, a PMS containing a smaller number of markers will undoubtedly save experimental costs. Thus, this study established a scheme to screen low-redundancy PMSs using the exhaustive algorithm and greedy algorithm. When screening PMSs, the greedy algorithm selects markers based on the parental dispersity index (PDI), a uniquely defined metric that outperforms the probability of exclusion (PE). With the conjunctive use of the two algorithms, non-redundant PMSs were found for more than 99.7% of solvable cases in three groups of random sample experiments in this study. Then, a low-redundancy PMS can be composed using two or more of these non-redundant PMSs. This scheme effectively reduces the number of markers in PMSs, thus conserving human and experimental resources and laying the groundwork for the widespread implementation of parentage assignment technology in economic species breeding.

## 1. Background

Parentage assignment can trace the parents of an offspring by the genotypes of molecular markers in them based on Mendel’s laws. It is commonly employed to differentiate families in forensics and economic species breeding, especially farmed fish breeding [1,2,3]. Economic fish usually lay large numbers of eggs [2], which are fertilized in vitro. This property makes it easy to artificially construct dozens or even hundreds of full-sib or half-sib families during a breeding season. For fish, if multiple families are reared separately, it is difficult to ensure that they are all in the same water and feeding conditions, so contemporary group effects may obscure family differences. In contrast, the polyculture of fish in the same pond and the differentiation of families through parentage testing would make the experiment more rigorous. Thus, it is an important application for fish breeders to accurately trace the parents of offspring based on the markers’ genotypes of the closed population and subsequently obtain accurate pedigree information for downstream analysis. Obviously, an efficient parentage marker set (PMS) with fewer markers can significantly reduce the cost of assigning thousands of offspring [4]. Moreover, fewer markers may improve the accuracy of parentage assignment [5].

The probability of exclusion (PE) [6,7], including the derived cumulative probability of exclusion (CPE) [8,9], is usually used to access the ability of marker(s) and filter PMSs. As the name suggests, PE is an estimate of the proportion of non-parents/non-parental pairs excluded by a single marker using the population genetic parameters. CPE can be thought of as the multiplication of PE values, indicating the exclusion ability of a set of markers. However, CPE cannot fully reflect the true power of multiple markers [1]. For example, if two linked markers both with high PE are adopted for parentage assignment, the CPE value will significantly increase but the actual parental exclusion ability does not improve. The software P-LOCI [10] was developed for this linkage problem to obtain a highly efficient PMS.

Obviously, the CPE does not take into account the interaction of parental exclusion ability among markers. The non-independent markers may falsely increase the CPE value, and then the cooperating parental exclusion effect of independent markers may also be ignored. The marker interaction information is discarded during the PE/CPE calculation. However, the marker cooperation effects are undoubtedly valuable for selecting of the most efficient marker combinations to reduce the cost of parentage assignment.

Other than PE, most parentage assignment software can also simulate numerous offspring genotypes based on candidate parent information to calculate the successful assessment rate of PMS [11]. Extending this strategy, if we simulate all possible genotyping combinations of the given markers across the offspring of all candidate parent pairs, theoretically, the smallest PMS that can determine all full-sib family parent pairs can safely be found by an exhaustive method of all marker combinations with a PMS size from 1 to all. A PMS that is just sufficient to distinguish all parent pairs is called a “non-redundant PMS” in the following section of the paper. The “minimum PMS” is the smallest one of all non-redundant PMSs for a population. In addition, if the given markers are not sufficient, the parental pairs and the corresponding simulated offspring with indistinguishable genotypes will also be found to guide the collection of new markers.

There are two key components to achieve the conception, i.e., a genotype matrix of all possible simulating offspring and a suitable algorithm to find non-redundant/low-redundancy PMSs. The genotype matrix of all simulated offspring is generated by using all potential parents’ correct genotypes of given markers, which are called full parental genotypes. For the artificial populations used in aquatic breeding, which often follow the factorial mating designs, such as the North Carolina (NC) II population, the candidate parent pools usually cover all potential parents, providing the possibility of simulating all possible offspring [4]. However, in natural populations, the candidate parent pools are usually samples of the entire adult population, making the full parental genotyping challenging.

On the other hand, the appropriate algorithm depends on the order of magnitude of the given markers. Different types of markers have different orders of magnitude. The currently widely used genetic markers for parentage assignment are microsatellite (also known as simple sequence repeat, SSR) [12,13] and single-nucleotide polymorphism (SNP) [3,14,15]. Due to the differences in detection methods and the polymorphisms, the size of a given SNP set, typically in the thousands, is always several orders of magnitude larger than that of an SSR set, which is typically less than a hundred. The exhaustive method can theoretically list all combinations of markers from the given marker pools and evaluate the parentage assignment capacity. However, there is no doubt that the number of marker combinations increases exponentially with the number of given markers, which in turn makes it impossible to obtain results in an affordable time. For the SNP sets that are always generated by next-generation sequencing with large sizes, the exhaustive method is not applicable, and the development of other low-complexity algorithms is needed.

Although a non-redundant PMS can be computed based on the above strategy, in actual testing, the marker genotyping issues, such as incorrect or missing genotyping, can seriously affect the success of parentage assignment. The robustness of a non-redundant PMS is undoubtedly poor. Merging multiple non-redundant PMSs into a low-redundancy PMS is an effective strategy to tolerate the genotyping problems and improve the success rate of parentage assignment.

Thus, we developed a PMS calculation software (v1.0.0) based on all simulated offspring genotypes from full parental genotyping, which combines exhaustive and greedy algorithms to adapt different sizes of the given marker set and implements the low-redundancy PMS computation centered on the minimum PMS members. The effectiveness of the two algorithms has been verified using simulated and real datasets. The software was developed in Python 3 and a user-friendly web version is also online (http://bioinfo.ihb.ac.cn/pmseeker, accessed on 28 January 2024).

## 2. Materials and Methods

### 2.1. Algorithm and Scheme

#### 2.1.1. Basic Definition

We define $P=\left\{P_{1},P_{2},\ldots ,P_{p}\right\}$ as the candidate parents and $P_{i}P_{j}$ (or $P_{i\times j}$) as the parent pair consisting of $P_{i}$ and $P_{j}$ and also their offspring set. $M=\left\{M_{1},M_{2},\ldots ,M_{n}\right\}$ is defined as the given marker set, and $G=(G_{i,j})$ is the allele vector (i.e., genotype) for the ith candidate parent on the jth marker. The genotype of parent $P_{i}$ on marker $M_{m}$ is denoted as $G_{i,m}=\{G_{i1},G_{i2}\}$, i.e., parent $P_{i}$ has two alleles $G_{i1}$ and $G_{i2}$. Analogously, the genotype of $P_{j}$ is denoted as $G_{j,m}=\{G_{j1},G_{j2}\}$. According to Mendel’s law of segregation, all possible genotypes of offspring of parent pair $P_{i\times j}$ are as follows:

$$G_{i\times j,m}=\{\left\{G_{i1},G_{j1}\right\},\left\{G_{i1},G_{j2}\right\},\left\{G_{i2},G_{j1}\right\},\{G_{i2},G_{j2}\}\}$$

The genotype set does not contain repeated elements, which means that the size of $G_{i\times j,\;m}$ varies from 1 to 4. For example, for a marker with 3 alleles (A, B, C), AA × BB gives AB, AA × BC gives AB, and AC, AB × AB gives AA, AB, and BB, while AB × AC gives AA, AC, AB and BC.

#### 2.1.2. Exhaustive Algorithm

The exhaustive algorithm for PMS screening must take into account all genotypes that various combinations of parents can theoretically produce, as well as all marker combinations of different sizes ranging from 1 to all, to determine whether the simulated offspring with any genotype vector can be completely traced back to the single parent pair according to Mendel’s laws. It traverses all the combinations ($C_{n}^{i}$) of *i* (*i* = 1, 2, 3, …, *n*) markers in this way and must compute $X=\sum_{i=1}^{n}C_{n}^{i}$ times. If a PMS has not been obtained, then it traverses the marker combinations of size *i* + 1 until all the combinations are exhausted (pseudo-code in Supplementary Section S4 Algorithm S1).

The PMSs produced by the exhaustive approach are referred to as “exhaustive-PMSs” in the following section of the study. The exhaustive algorithm outputs only non-redundant PMS(s) in ascending order of the PMS size. The first output must be the global best solution (the minimum PMS) and there may be multiple minimum PMSs. The PMSs checked by the exhaustive algorithm can definitively distinguish every offspring when the genotyping errors in the experiment operations are not taken into account. If there is no solution, the program will also output the indistinguishable parent pairs to guide the addition of new markers.

#### 2.1.3. Multiway Tree-Based Greedy Algorithm

In order to describe the tree structure in detail, *N* represents the node, and $N_{l}=\{N_{l,\;1},N_{l,2},\ldots \;N_{l,n}\}$ represents all nodes at the *l*-th layer, where $N_{l,i}$ represents the *i*-th node in the *l*-th layer. $S_{l,i}$ is used to store the set of parent pairs in node $N_{l,i}$, $n_{l,i}$ is used to denote the number of parent pairs in the node $N_{l,i}$, and $T_{l}$ denotes the number of nodes contained in the *l*-th layer. When $S_{l,i}$ contains multiple parent pairs, $N_{l,i}$ is called a branch node; if there is only one parent pair in $S_{l,i}$, $N_{l,i}$ is a leaf node.

Theoretically, the algorithm will converge quickly if all sets of parent pairs in the same layer are similar in size. Therefore, we used the sum of the mean and standard deviation to measure the degree of dispersion of parent pairs (Parental Dispersion Index, PDI). The calculation formula is as follows:

$${PDI}_{m}={mean}_{m}+{SD}_{m}$$

where

$${mean}_{m}=\frac{1}{T_{\left(l+1\right)}}\sum_{i=1}^{T_{\left(l+1\right)}}n_{\left(l+1\right),i}$$

$${SD}_{m}=\sqrt{\frac{1}{T_{\left(l+1\right)}}\sum_{i=1}^{T_{\left(l+1\right)}}{\left(n_{\left(l+1\right),i}-{mean}_{m}\right)}^{2}}$$

The PDI of marker $M_{m}$ is the sum of the average and standard deviation of the number of parent pairs in the remaining child nodes $N_{\left(l+1\right),i},$ excluding all leaf nodes and repeated branch nodes that contain the same set of parent pairs after all parent pairs in $N_{l}$ are genotyped by the marker $M_{m}$. The smaller PDI indicates the more nodes generated by parent pairs in $N_{l}$ and the smaller difference in the number of parent pairs in each node of $N_{\left(l+1\right)}$, which means that the marker has a better effect in distinguishing $N_{\left(l+1\right),i}$.

A specific marker will finally differentiate all parent pairings after layer-by-layer screening. It has now reached the bottom of the tree structure, where each node is a leaf node and there are no more branch nodes, and the PDI has now reached its minimal value of 0.

**Figure 1:**
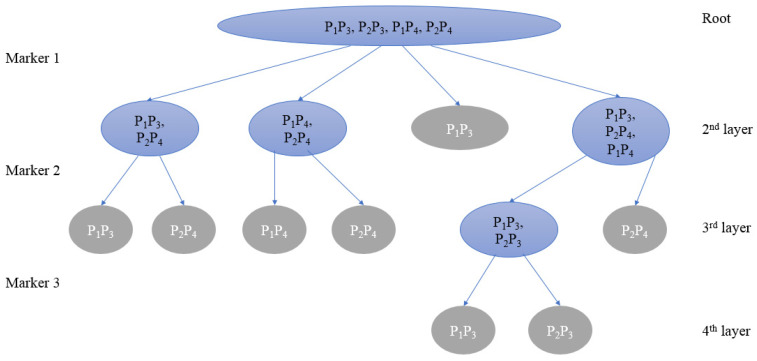
Schematic diagram of the greedy algorithm. Among them, the blue node is the parent pair to be distinguished, namely the branch node; the gray node is the leaf node.

The program screens markers by creating a multiway tree. All candidate parent pairs make up the root node. With the first layer of the tree, different markers can be used to produce the nodes of the next layer of the tree. The optimal next layer is chosen as the new layer with the lowest PDI, and the associated marker is added to the final PMS. By looping in this manner until all child nodes of some layer are leaf nodes, the whole multiway tree is retrieved, and the local optimal PMS is obtained (pseudo-code in Supplementary Section S4 Algorithm S2 and Figure 1). Additional PMSs can be generated after removing one or more PMS markers from the given marker set and reapplying the greedy algorithm on the shrunken set. In the following section of the paper, PMSs obtained by the greedy algorithm will be referred to as “greedy-PMSs”, which are usually of very low redundancy.

#### 2.1.4. Scheme to Constitute a Low-Redundancy PMS

By utilizing the non-redundant or very low-redundancy PMSs generated through exhaustive and/or greedy algorithms, a new PMS with low redundancy can be constructed simply by combining the first two output PMSs. The number of PMSs to be merged can also be artificially adjusted according to the experimental needs.

### 2.2. A Comparative Study on the Running Time of the Two Methods

We simulated mixed populations of 5 various sizes and utilized SNP markers, the most prevalent dimorphic marker in the genome, to generate given marker sets in order to compare the efficacy of the two algorithms with varied numbers of given markers and families. Each set was made up of the optimal SNP sets and background markers. The sets with a modest number of markers (>20) are used to investigate the efficacy of the two algorithms, while the sets with a large number (>100) are used to investigate the greedy algorithm. Specific simulation and operating methodologies are detailed in Supplementary Section S1.

### 2.3. PMS Screening Based on Real Molecular Markers

Microhaplotype marker is a novel genetic marker consisting of a group of adjacent SNPs and has potential applications in parentage assignment [16,17]. To better capture genome-wide marker properties, we chose 225 microhaplotype markers dispersed across the grass carp genome [18] as the given marker set (dubbed “MF2016 Marker Set”). These markers were genotyped in five grass carp parents, which were used to generate an NCII population (3♂ × 2♀) in 2016. We screened the PMSs for this closed population and analyzed the performance of both algorithms independently under the conditions of known and unknown gender of the candidate parents.

We conducted three different experiments on the 225 markers, each with 1000 groups of given markers, each with 10 markers randomly picked from the MF2016 Marker Set. The greedy-PMSs were compared in terms of success rate, result agreement rate, number of markers, and associated statistics, with the exhaustive-PMS being used as the optimal solution.

### 2.4. Application in a Real Case

To investigate to what extent genotype errors and missingness affect the parentage assignment efficacy of greedy-PMSs, we used the practical datasets of the Mexican gray wolf from Andrews, Adams, and Cassirer [19], the only applicable study we could find. These datasets provide (1) authentic correspondences of parent–child trios and (2) authentic genotypes and observed genotypes of both SSR and SNP markers. After generating PMSs from the marker sets, parentage testing was performed using CERVUS 3.0 [20] and COLONY 2.0 [21], respectively, and the results were filtered using specified criteria according to the application scenarios of the program. Supplementary Section S3 contains the detailed procedures.

## 3. Results

We evaluated the running times of the two algorithms using simulation data and analyzed the PMSs obtained by the two algorithms using markers from the real genome. To evaluate the greedy-PMSs’ performance and to identify potential difficulties in actual applications, we used real data from Mexican gray wolves for parentage assignment.

### 3.1. A Comparative Study on the Running Time of the Two Algorithms

Apparently, the computational effort is determined by the number of given markers and the number of families involved. We set the running time threshold to 5000 s, which is sufficient to show the change in each algorithm, and then generated a series of datasets in silico to test the time efficiency of the algorithms in scenarios with different numbers of markers and families (Supplementary Section S1). The results revealed that the exhaustive algorithm was constrained by the quantity of given markers. The running time (>5000 s) expired when the number of given markers reached 19 and the family number reached 10. The greedy method, on the other hand, has no restriction on the number of given markers. When the number of given markers was low, the exhaustive algorithm outperformed the greedy algorithm. However, after the number of given markers reached 17, the greedy algorithm began to outperform in terms of speed. In fact, the greedy algorithm’s running time was proportional to the number of markers (Figure S1 in Supplementary Section). As a result, even with a huge number of markers, the greedy algorithm can screen out PMS quickly.

### 3.2. PMS Screening Based on Real Molecular Markers

#### 3.2.1. Summary of the Solution Acquisition Rate

When compared to simulated data, real data typically contain more complicated noise, which can reduce the efficacy of screened markers. Thus, we employed 225 genuine molecular markers from the MF2016 marker set in three concurrent studies, each sampling 1000 sets of markers as given marker sets. Parental gender information is frequently overlooked throughout the reproduction process, resulting in an increase in the potential number of families and, ultimately, affecting the required PMS. In order to match the practical application, we performed PMS screening on the MF2016 marker set with and without parental gender information. Using exhaustive-PMSs as the gold standard, we investigated the greedy algorithm’s PMS-screening efficacy under various situations.

For clarity, we used the following definitions: The minimum PMS is called an optimal solution. A solution with a greedy-PMS that partially overlaps with any optimal solution is called an intersection solution. A solution that includes any non-redundant PMS as a subset is a redundant solution. In all simulated datasets of known and unknown genders, the greedy algorithm could also find a solution where the exhaustive algorithm had a solution (Table 1). However, the greedy-PMS can be a non-redundant solution or a low-redundancy solution. If a redundant PMS is obtained, the exhaustive algorithm can be used with it to remove redundant markers and obtain a non-redundant PMS. After de-redundance, the optimal solution rate of the greedy algorithm reached 99.71%.

#### 3.2.2. Comparison of the Efficacy of PDI, PIC, and PE

Using the same datasets, we calculated the PDI, PIC, and PE of 225 real molecular markers (Supplementary Section S3 details the calculation method), as well as the relationship between the three indexes. The lack of parents’ gender information made the genotype combinations of simulated offspring more complex and the PMS more difficult to find, but regardless of gender, the association between PIC and PE was very strong and both were inversely related to PDI (Table 2), with the correlations between the three being highly significant (*p* < 2.2 × 10^−16^).

**Figure 2:**
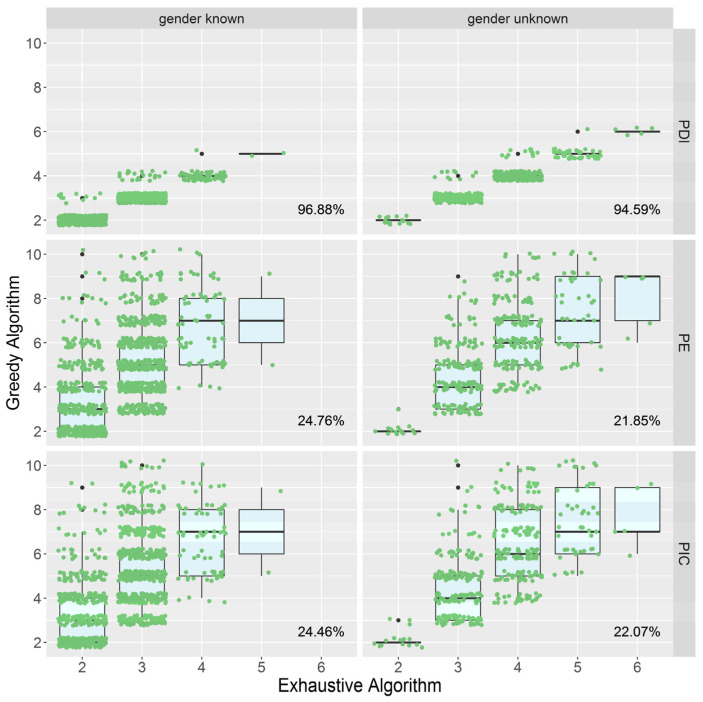
The distribution of the number of markers in PMSs screened with PDI, PE, and PIC.

To examine the efficacy of PMS screening utilizing the greedy algorithm with PDI, PIC, and PE as indicators, we employed the above randomly sampled data to screen the PMS. The number of markers in PMSs acquired by PE and PIC is three to five times that of PMSs obtained by PDI, and the range of variance is wider (Figure 2). In consequence, as compared to PIC and PE, utilizing PDI as the indicator can remove redundant markers more effectively and achieve a more optimized PMS.

The horizontal axis represents the number of markers contained by the exhaustive method (the optimal solution), and the vertical axis represents the number of markers produced by the greedy algorithm. The cases of known gender are shown on the left, and the cases of uncertain gender are shown on the right. The data in the lower right corner of each figure is the rate of obtaining the optimal solution under the current situation. For example, “96.88%” indicates that when the gender is known, the rate of achieving the optimal solution is 96.88% using PDI as the greedy algorithm’s indicator.

In the PMSs generated by two algorithm sets with and without gender information, we estimated the average PIC and CPE values of PMSs, which are used to assess the polymorphism and effectiveness of parentage assignment for a certain marker set (Table 3). According to the results, the number of markers in greedy-PMSs and the number of markers in minimum PMSs are approximately the same. The average PIC of the PMSs obtained by the two algorithms was approximately 0.61, and the CPE was approximately 0.91 (gender known) and 0.97 (gender unknown).

### 3.3. Application in a Real Case

With the use of the dataset from Andrews, Adams, and Cassirer [20], we were able to determine the accuracy of parentage assignment in real circumstances by employing greedy-PMSs. We screened two greedy-PMSs and combined them to generate the third PMS as a low-redundancy marker set (methods in Supplementary Section S3). These three sets of PMS were used to conduct parentage assignment through CERVUS 3.0 and COLONY 2.0, respectively (Table 4).

Both CERVUS and COLONY correctly identified the parents for all offspring without problematic markers, indicating a high effectiveness of the greedy-PMSs. Using SSR PMSs, these two tools also achieved very good accuracy rates in the offspring with one false or missing marker, and only two offspring were not assigned parents by CERVUS (PMS “S1-1”). As for SNP PMS, genotype errors showed a significant negative impact on parentage assignment. When the size of a greedy-PMS did not exceed 6, both tools failed with a single false marker in most cases, with the only exception that COLONY succeeded on PMS “45-2”.

Using a low-redundancy PMS pooled from two greedy-PMSs, the test was successful for all but three cases with incorrect parents assigned: an offspring with one erroneous marker out of nine markers in PMS “1-1+2” (only CERVUS failed), and one offspring missing half the markers in PMS “45-1+2” (both tools failed). These results suggest that modestly increasing the redundancy of a PMS can effectively eliminate the negative impact of missing genotypes and genotype errors.

### 3.4. The PMSeeker Online Tool for PMS Screening

The process of PMS screening has been implemented as an online tool, PMSeeker (http://bioinfo.ihb.ac.cn/pmseeker, accessed on 28 January 2024), which is freely available to the public. A simple and clear interface is provided for users to upload data and set parameters. In particular, the default option for the algorithm is “Recommended”, which means that the greedy algorithm will be applied first to obtain a greedy-PMS, and if applicable, the exhaustive algorithm will be further used on the greedy-PMS to remove possible redundant markers. The results include a base PMS and a set of recommended redundant markers from other PMSs, no more than twice as many as in the base PMS. Users can use the base PMS and any number of markers in the redundancy set to form a low-redundancy PMS.

## 4. Discussion

As a non-redundant/low-redundancy PMS calculation software, PMSeeker (v1.0.0) uses full parental genotypes to simulate the whole offspring genotype combinations for the PMS filtering. It implements two algorithms: the exhaustive one for small given marker sets such as SSRs, and the greedy algorithm for huge given marker sets such as SNPs. Here, we evaluated the attributes of the two algorithms and discovered some concerns that need to be considered in real-world applications during the study process.

### 4.1. The Advantages and Disadvantages of the Two Algorithms and the Efficacy of Their Combination

To find the optimal sets of markers, the exhaustive method goes through all possible marker combinations and all conceivable (or provided) parent pairs one by one. The computational complexity reaches O(N!∗p), where *p* is the number of all possible families, and *N* is the number of given markers. *N* is the main factor that causes the exponential growth in computational complexity. As a result, the exhaustive algorithm is unsuitable for scenarios with a large number of given markers. When N = 19 in our test, the running time of virtually all simulations exceeded 5000 s (Supplementary Section S1).

Additionally, the greedy algorithm screens the layer’s local optimal marker based on PDI. Each optimal solution must cross all the markers a maximum of *M* times. That is, the computational complexity is O(M∗N∗p), where *M* denotes the total number of markers contained in the final PMS. During the actual computation process, when the tree structure is constructed, the number of parent pairs to distinguish in each child node of the new layer is smaller than the number of original parent pairs. Meanwhile, the greedy algorithm merely needs to filter the remaining potential markers one by one to find the marker with the lowest PDI. The two aspects outlined above significantly minimize the time required for calculation while screening PMSs. Due to the advantages listed above, the greedy algorithm is well suited for scenarios involving a high number of given markers.

In contrast to the exhaustive method, the greedy algorithm seeks the local optimal solution, with an optimal solution rate of more than 90% (Table 1). After screening the greedy-PMS with the exhaustive approach, the rate increased to 99.7%, demonstrating that the combination of the two methods is more efficient than utilizing the greedy algorithm alone in obtaining the ideal PMS. Although this is not as good as utilizing the exhaustion algorithm exclusively, this combination is unquestionably a strong strategy in many circumstances where the number of given markers collected from the entire genome is large and the exhaustive algorithm cannot be employed directly.

### 4.2. Comparison of the Statistics Used in PMS Screening

Currently, screening procedures for PMS can be loosely classified into two groups based on the sorts of markers. For molecular markers with medium and high polymorphisms, molecular markers with high polymorphisms are recommended as much as possible. Thus, statistics referring to polymorphisms, such as PIC [22,23] and PE [7], are utilized, which primarily indicate the discriminability of a single marker for individuals. For low-polymorphism SNP markers, it is primarily to eliminate comparable markers (based on linkage disequilibrium) and markers that are not sufficiently authentic (based on Hardy–Weinberg balance, MAF, etc.).

With the application backdrop of parentage assignment in mind, we created a statistic, PDI, that is utilized to represent the discriminability for candidate parent pairs of one marker. The greater the discriminability, the lower the value. The relationship between PDI, PIC, and PE of 225 markers in the real marker collection was compared (Table 2). The findings revealed that PIC and PE were substantially positively related (r = 0.988), but PDI was negatively and less correlated with the two (r ≈ −0.8). The correlation between PDI and PIC/PE was stronger in the absence of parental gender information, indicating that when parental gender information is missing, PDI is closer to distinguishing individuals, and higher polymorphic markers may be required to separate parent pairs. This makes sense: a lack of parental gender information will increase the number of families, where appropriate inclusion of highly polymorphic markers is conducive to obtaining PMSs.

PDI evaluates the degree (the average value of the parent pairs in each subgroup) and the uniformity of division (standard deviation) when a marker divides one group of parent pairs into numerous subgroups, directly and precisely measuring the discriminability of the marker. As a result, the optimal solution obtaining rate with PDI as the indicator is substantially greater than the rate with PIC and PE (Figure 2), implying that the greedy algorithm has a significantly stronger de-redundancy ability with PDI as the indicator than with PIC and PE. In conclusion, as compared to PIC and PE, PDI can further reduce the number of markers found in PMS and is better suited for screening PMS.

The key indicators utilized in parentage assignment for a PMS include the overall average PIC, CPE of the marker set, and so on. On the basis of this, we investigated the distribution of these markers in the aforementioned resoluble sets (Table 3). In published papers, the polymorphisms of PMS are usually high (about 0.7), and the CPEs are frequently above 0.999 [8,24,25,26,27,28,29]. The average PIC of PMSs obtained by our approach, however, is about 0.61, and the CPE value is less than 0.97. The primary explanation for this disparity is that we acquired considerably fewer markers in the PMS (approximately 3) than those in other studies (all above 10), making the calculation of CPE more vulnerable to markers with lower PE. In the real dataset, a similar result is also displayed (Table 4). This demonstrates that whether the CPE value is large enough does not fully correspond to how successful the PMS is, as Vandeputte, Rossignol, and Pincent [1] described.

### 4.3. Effectiveness, Problems, and Solutions in Practical Applications

Ideally, a non-redundant PMS or very low-redundancy greedy PMS would be sufficient to accomplish parentage assignments in a given population at a very low cost. However, the reality is always far more complex; missing genotypes and genotyping errors are often unavoidable and must be carefully considered. Naturally, redundant markers are needed to handle this situation. Then, how many and which markers to use remains a question. That is why we propose this low-redundancy PMS scheme based on exhaustive-PMS and/or greedy-PMS.

It is important to recognize that PDI-based PMS calculations may not be suitable for markers with ungenotyped sample(s) in the parental pool. However, when it comes to SNPs, parental genotypes are consistently obtained from high-throughput sequencing, and the abundance of SNPs enables the acquisition of a sufficient number of candidate markers without encountering genotyping failures. Moreover, in the case of SSRs, the experimental remediation of several missing marker genotypes can be achieved at a reasonable cost. This further enhances the feasibility of employing our method in parentage assignment studies.

#### 4.3.1. The Efficacy of the Low-Redundancy Scheme

To assess the efficacy of our scheme in actual applications, we searched public data for datasets with known parent–child correspondences and the genotypes of SSR/SNP markers in each individual [5,8,20,26,30,31,32,33] and found only one study [19] that met all the requirements. Thus, we used five datasets from this study for testing. As shown in Table 4, our scheme significantly reduced the number of markers included in a PMS, typically screening three to six markers from hundreds of candidates as a greedy-PMS. This is low even when doubling the number of markers in a low-redundancy PMS. Such a small PMS will vastly reduce the cost of parentage assignments.

As we expected, every parent–child relationship was correctly identified using a single greedy-PMS if all involved markers were correctly genotyped. Using greedy-PMSs, CERVUS and COLONY failed on most offspring with genotype errors and a small percentage of cases with missing genotypes (Table 4). However, if low-redundancy PMSs based on two greedy-PMSs were used, only CERVUS failed on an offspring with one genotype error, and both tools failed on an offspring with six missing genotypes. These results indicated that the low-redundancy PMS scheme can well balance the cost and performance of parentage assignment. In fact, using a small number of markers brings additional advantages, for example, we can have a wider choice of genotyping instruments, and perhaps older non-high-throughput assays (such as Sanger sequencing) can be used even more to facilitate more accurate genotyping of SSR/SNP markers.

#### 4.3.2. The Different Impacts of Two Types of Problematic Markers

We evaluated the effect of missing genotypes and genotype errors on the accuracy of parentage assignments. As mentioned above, parentage assignments with a single greedy-PMS failed in most cases involving even just one genotype error. But the situation with missing genotypes is obviously different. It is not uncommon for a test to succeed in the absence of a small number of markers in a greedy-PMS, while many failures are caused by the loss of multiple markers, even as much as half of the PMS (Table 4). It is clear that genotype errors are more likely to produce failures, whereas missing genotypes have a much smaller influence than genotype errors. This fact suggests that we should employ stricter criteria when genotyping markers to reduce erroneous genotypes. Measures that can be considered to reduce genotype errors include the following: For SSR markers, it is recommended to carefully check the genotype of each marker or use the leave-one-out method to troubleshoot conflicting markers in the PMS; for SNP markers, it is recommended to use targeted sequencing or an SNP array to directly perform accurate SNP genotyping.

#### 4.3.3. The Different Efficacies of SSR and SNP

The characteristics of the marker will also have an impact on our screening scheme for PMS. Genotyping error of PMS markers has a significant impact on the efficacy of parentage assignment. The effect of allelic error varies by marker type. A single allelic error has less of an effect on SSRs that have multiple complex alleles than on SNPs, which are mostly dimorphic.

In our tests, the two SSR greedy-PMSs and the low-redundancy PMS consist of 3, 4, and 5 markers, respectively. While CERVUS was unable to assign parents to the two offspring with incorrect genotypes using the three-marker PMS (“S1-1”), it succeeded when using either the four- or five-marker PMS. This result implied that simply adding a few SSR markers can greatly improve fault tolerance. For the sake of reliability, we suggest that a low-redundancy SSR PMS should be constructed by supplementing the base greedy-PMS or exhaustive-PMS with half its number of additional markers.

For SNP, an allele error may completely reverse its meaning and role in identification, leading to erroneous conclusions. As we saw in Table 4, one genotype error in the six-marker greedy-PMSs was misleading in almost all cases, but the low-redundancy PMSs with twice the markers effectively diminished its negative effect. Therefore, we propose that the low-redundancy SNP PMS should contain twice as many markers as the base greedy-PMS or exhaustive-PMS.

Given the devastating impact of SNP genotype errors on the success rate of parentage assignment, ensuring the accuracy of SNP markers is paramount. The marker’s own MAF value can be utilized to filter some SNP sites with random genotyping errors in the targeted population. The higher the MAF, the more accurate the SNP loci obtained. We recommend that the SNP loci with genotypes missing in sequencing data be eliminated and a significant number of potentially erroneous markers be removed by filtering MAF values and using other approaches [33]. Following that, the remaining high-accuracy SNP markers can be utilized to screen PMS using the greedy algorithm, followed by the exhaustive algorithm.

#### 4.3.4. About the Software Tools for Parentage Assignment

Although this study was designed to screen for low-redundancy PMSs, which do not rely on certain parentage assignment software tools, it is instructive to see if there is evidence that low-redundancy PMSs perform better with some specific software. While there are a number of tools for assigning parentage using genetic markers, we do not have many options for our testing, as most of them either used obsolete types of markers or are no longer maintained, for example, PAE [34], Sequoia [35], and FAP [36]. In particular, FAP seems suitable for our application scenario, as it can identify marker loci that are consistently associated with parental allocation errors and should be removed from the PMS. Unfortunately, we could not find any download sources or even contact the authors. Therefore, we used CERVUS 3.0 and COLONY 2.0, the two most commonly used tools.

In our tests, COLONY performed slightly better with erroneous markers, but CERVUS had one less failure due to missing markers. In fact, it is hard to simply say which is better. CERVUS (version 3.0) is a pairwise likelihood-based software for single-dyad assignment, while COLONY takes into account the likelihood of an entire pedigree configuration (multiple dyads). Accordingly, COLONY is more computationally demanding and more efficient in using genetic markers than CERVUS [22,37]. Therefore, we suggest that COLONY is often a better choice for parentage assignments to a small number of offspring based on PMSeeker-generated markers. Additionally, since COLONY tends to specify unsampled parents if the sampled parents do not match some cluster of some offspring [37,38], the option “candidate parents are all sampled in the pool” should be set for the closed population with PMSeeker applied. If the number of offspring is very large (>10,000), COLONY may take much more time (3 days vs. 30 min, Karaket and Poompuang [39]) and CERVUS is recommended.

### 4.4. Scalability of the Scheme

The scheme developed in this study was initially designed to minimize the number of markers used in parentage assignment in fish breeding under the conditions that (1) the parents together with offspring make up a closed population, (2) all candidate parents have been genotyped, and (3) the species applying this scheme are diploid. So, it is possible to be utilized in PMS screening for a wider range of species as long as the scenario meets the conditions mentioned above. For instance, parentage assignment has been widely used in diploid livestock breeding [5,8,19,24,32,40,41], where repeated use of artificial insemination leads to inaccurate pedigree records [42]. So, the scheme developed in this study has the ability to reduce the cost of parentage assignment, ensuring accuracy, in the same way as the fish breeding.

## 5. Conclusions

An innovative online software, PMSeeker, has been developed to facilitate the process of parentage assignment. PMSeeker utilizes complete parental genotypes to simulate various combinations of offspring genotypes for PMS filtering, with the goal of obtaining non-redundant or low-redundancy PMS(s). Depending on the size of the given marker set, PMSeeker employs two algorithms: the exhaustive algorithm for smaller sets and the greedy algorithm for larger sets. The exhaustive algorithm, although computationally slower, ensures the acquisition of the best possible PMS(s). On the other hand, the greedy algorithm significantly enhances computational speed, albeit with a slight compromise in the likelihood of obtaining the absolute best PMS(s). However, regardless of the algorithm used, the non-redundant or low-redundancy PMS obtained through PMSeeker consistently demonstrates excellent accuracy in parentage assignment, as evidenced by both simulated and real data. Overall, PMSeeker presents a valuable tool for researchers and practitioners in the field, offering efficient and reliable parentage assignment results through its advanced computational algorithms.

## Figures and Tables

**Table 1:** Summary of resoluble set rate and optimal solution obtaining rate with and without gender information.

Gender	Group	Both Algorithms		Greedy Algorithm				
		ResS Number	ResS Rate (%)	OS Number	OS Rate (%)	RedS Number	Rate of OS and RedS (%)	IS Number
Known	1	326	32.6	319	97.85	4	99.08	3
	2	349	34.9	334	95.7	15	100	0
	3	351	35.1	341	97.15	10	100	0
	Sum	1026	34.2	994	96.88	29	99.71	3
Unknown	1	146	14.6	142	97.26	4	100	0
	2	145	14.5	139	95.86	5	98.62	1
	3	153	15.3	139	90.85	14	100	0
	Sum	444	14.8	420	94.59	23	99.77	1

Note: This test is based on simulated data and 225 genuine molecular markers from the MF2016 marker set of grass carp. Abbreviations in the table are as follows: ResS, resoluble set; OS, optimal solution; RedS, redundant solution; IS, intersection solution.

**Table 2:** Spearman correlation coefficients between statistics when the parents’ gender was known or unknown.

	*r* (Gender Known)	*r* (Gender Unknown)
PIC vs. PE	0.9886	0.9886
PDI vs. PIC	−0.7783	−0.8738
PDI vs. PE	−0.7913	−0.8766

Note: This test is based on the same data used for Table 1.

**Table 3:** Summary of the indicators of PMSs in resoluble sets (mean ± SD).

Indicators	Gender Known		Gender Unknown	
	Exhaustive	Greedy	Exhaustive	Greedy
Marker number	2.6686 ± 0.6051	2.6998 ± 0.6321	3.6306 ± 0.7593	3.6847 ± 0.7920
Average PIC	0.6012 ± 0.0844	0.6141 ± 0.0823	0.6138 ± 0.0739	0.6231 ± 0.0711
CPE	0.9078 ± 0.0507	0.9143 ± 0.0478	0.9665 ± 0.0214	0.9698 ± 0.0180

**Table 4:** Summary of the parentage assignments for Mexican gray wolf (data set from Andrews [19]) using CERVUS 3.0 and COLONY 2.0 in the case of genotype error and genotype missing.

PMS ^†^	Number/Candidate Number	Average PIC	CPE	CERVUS Accuracy Rate (%)	COLONY Accuracy Rate (%)	Failures on Genotype Error ^‡^			Failures on Missing Genotype ^§^		
						Offspring	CERVUS	COLONY	Offspring	CERVUS	COLONY
S1-1	3/22	0.669	0.9611	16 (88.89)	18 (100.00)	2	2	0	0	-	-
S1-2	4/22	0.6754	0.9871	18 (100.00)	18 (100.00)	1	0	0	1	0	0
S1-1+2	5/22	0.6817	0.9962	18 (100.00)	18 (100.00)	2	0	0	1	0	0
1-1	4/999	0.3023	0.6613	14 (82.35)	13 (76.47)	3	3	3	3	0	1
1-2	5/999	0.3309	0.7705	17 (100.00)	17 (100.00)	0	-	-	2	0	0
1-1+2	9/999	0.3182	0.9223	16 (94.12)	17 (100.00)	3	1	0	5	0	0
4-1	6/326	0.3682	0.8573	15 (88.24)	14 (82.35)	1	1	1	5 (3) ^¶^	1 (3)	2 (3)
4-2	5/326	0.3652	0.8000	17 (100.00)	17 (100.00)	0	-	-	3	0	0
4-1+2	11/326	0.3668	0.9714	17 (100.00)	17 (100.00)	1	0	0	6 (4, 2)	0	0
45-1	6/201	0.3690	0.8587	14 (82.35)	15 (88.24)	1	1	1	5 (3)	2 (3)	1 (3)
45-2	6/201	0.3733	0.8609	15 (88.24)	16 (94.12)	1	1	0	4 (3)	1 (3)	1 (3)
45-1+2	12/201	0.3712	0.9802	16 (94.12)	16 (94.12)	2	0	0	6 (6, 2, 2)	1 (6)	1 (6)
475-1	6/111	0.3662	0.8557	16 (94.12)	16 (94.12)	1	1	1	4 (3)	0	0
475-2	6/111	0.3705	0.8589	17 (100.00)	17 (100.00)	0	-	-	4 (3)	0	0
475-1+2	12/111	0.3719	0.9273	17 (100.00)	17 (100.00)	1	0	0	6 (4, 3, 2)	0	0

Note: ^†^. PMS S1 corresponds to the dataset MSAT, while PMSs 1, 4, 45, and 475 correspond to the datasets MAF1 (MAF > 0.1), MAF4 (MAF > 0.4), MAF45 (MAF > 0.45), and MAF475 (MAF > 0.475), respectively. ^‡^. For CERVUS 3.0, except PMS 1-1+2 for which CERVUS returned a wrong parent pair for one offspring (MGW_1352), all identification failures were that CERVUS could not identify an offspring to any parent pair. For COLONY 2.0, except PMS 1-1 for which COLONY returned wrong parent pairs for MGW_1352 and MGW_1354, failures were all attributed to the inability to identify any parent pair for MGW_1346. ^§^. All identification failures in this column were that the best parent pair identified by both CERVUS and COLONY was not the true parent of the offspring. ^¶^. The number in parentheses indicates the number of markers with genotype errors/missing in one offspring (number 1 is omitted). Here, “5(3)” means that each of the 5 offspring has 3, 1, 1, 1, and 1 marker(s) with genotyping problems, respectively.

## Data Availability

The algorithm has been implemented at http://bioinfo.ihb.ac.cn/pmseeker, accessed on 28 January 2024, and the data used in this article are available from Dryad with a DOI (https://datadryad.org/stash/share/ZiqKI3LqxksiFEVlnOD7DmPgA0O2_eO3XsfE7eTjOEg, accessed on 28 January 2024).

## Supplementary Materials

The following supporting information can be downloaded at https://www.mdpi.com/article/10.3390/biology13020100/s1. The Supplementary Material file contains information about the comparative study on the running time of the two algorithms, the application in a real case, and the analysis of the polymorphism and exclusion probability.
