# Supplementary material for: PMSeeker: A Scheme Based on the Greedy Algorithm and the Exhaustive Algorithm to Screen Low-Redundancy Marker Sets for Large-Scale Parentage Assignment with Full Parental Genotyping
Source: Biology (Basel). 2024 Feb 5;13(2):100. doi: 10.3390/biology13020100 (PMC10886308; doi:10.3390/biology13020100)
Supplement: Supplementary file 1 [file biology-13-00100-s001.zip › biology-2823580-supplementary.pdf]

PMSeeker: a scheme based on the greedy algorithm and the exhaustive algorithm to screen low-redundancy marker sets for parentage assignment of diploid species

Lei Xia, Heng Li, Wanting Zhang, Yingyin Cheng, Mijuan Shi, Xiao-Qin Xia

Section S1: A Comparative Study on the Running Time of the two algorithms

1.1 Methods

Simulation data generation method

In order to explore the efficiency of the exhaustive algorithm and the greedy algorithm for different number of candidate markers and families, we generated families (parent pairs) and candidate markers through simulation. Families were generated in the form of one-to-one correspondence between parents. The marker sets were constructed as SNP markers, the most common dimorphic markers in the genome, including the optimal SNP marker sets and some background markers. For the optimal SNP marker sets, it was required that: (1) the markers in the combination were homozygous in each parent (i.e., "a/a" or "b/b"); (2) according to the offspring's genotypes ("a/a", "a/b", "b/b"), each marker could divide each group of parent pairs evenly into 3 subgroups, and each parent pair could only appear in one subgroup. Based on the above two requirements, we generated each optimal marker iteratively until the genotypes of each parent pair were unique, which means that we generated $\lceil log_3 p \rceil$ markers where p stands for the number of parent pairs. Through the optimal SNP marker sets generated, simulation of parents' genotypes was performed according to Mendel's law of separation. For the background markers, the genotypes of dimorphic SNPs were randomly generated for each parent. Finally, a candidate marker set was generated by combining the optimal marker set and the background marker set.

Comparative study of running time

In a test, *m* (*m*=5, 7, 9, 11, 13, 15, 17) candidate markers were randomly selected to test the exhaustive algorithm and the greedy algorithm in a population of *n* (*n*=10, 30, 50, 75, 100) parent pairs. The test was repeated 5 times at each point (*m*, *n*). A running would be terminated if the running time was longer than 5000s, and the algorithm was considered unable to obtain PMS under the test conditions if no less than 3 out of the 5 repeated tests were not completed.

In order to further test the PMS detection efficiency of the greedy algorithm with a large number of candidate markers, we adopted bigger *m* values (*m*=100, 1000, 2500, 5000, 10000) to test the greedy algorithm. Thus, a total of 25 PMS screening experiments were performed in the above five populations, and each experiment was repeated 100 times.

We also simulated the situation when PMS did not exist. The genotype was set to "a/a" for the first 50 parent pairs in the optimal sets, and "b/b" for the other half parent pairs, then some background markers were added to form the final non-solution set.

1.2 Results

In order to explore the time efficiencies of the two algorithms, we compared their running time for PMS screening through the dimorphism markers and candidate parent pairs generated by simulation. As shown in the results, when the number of candidate markers reached 19 and the number of parent pairs reached 10, it took an average of 1271s using the exhaustive algorithm. On this basis, increasing the number of markers or the number of parent pairs resulted in a timeout (>5000s). Therefore, for ease of description, we only selected the results with candidate markers within 17 for display (Figure 1A in Supplementary Section). Although the exhaustive algorithm was slightly faster than the greedy algorithm for fewer markers, when the number of candidate markers increased to 17, the greedy algorithm began to show speed advantages.

While the exhaustive algorithm was not suitable for a large number of candidate markers, the running time of the greedy algorithm was roughly linearly related to the number of markers and the number of families (Figure 1B-C in Supplementary Section). The largest test dataset contained 10000 markers and 100 families, and the average time

consumed by the greedy algorithm was 4953.38s, and all simulations was completed within 5000s.

In summary, for dimorphic SNP markers, when the number of candidate markers was less than 15, the exhaustive algorithm was better, but when the number of markers was large, the greedy algorithm was the best or even the only option. The simulations above were implemented with the following configurations: CPU: Intel(R) Xeon(R) CPU E7-4820 v2 @ 2.00GHz, RAM: 1.0 TB.

As for the SSR marker, we did not test the efficiency as above, because it is a kind of codominant marker which is hard to simulate due to the high polymorphism. According to what we've tested elsewhere, when it came to 4000 candidate markers and 150 candidate families in a closed population, the average time exceeded 5000 s. Subsequently, we limit the number of markers (~10000) and families (~100) in our website.

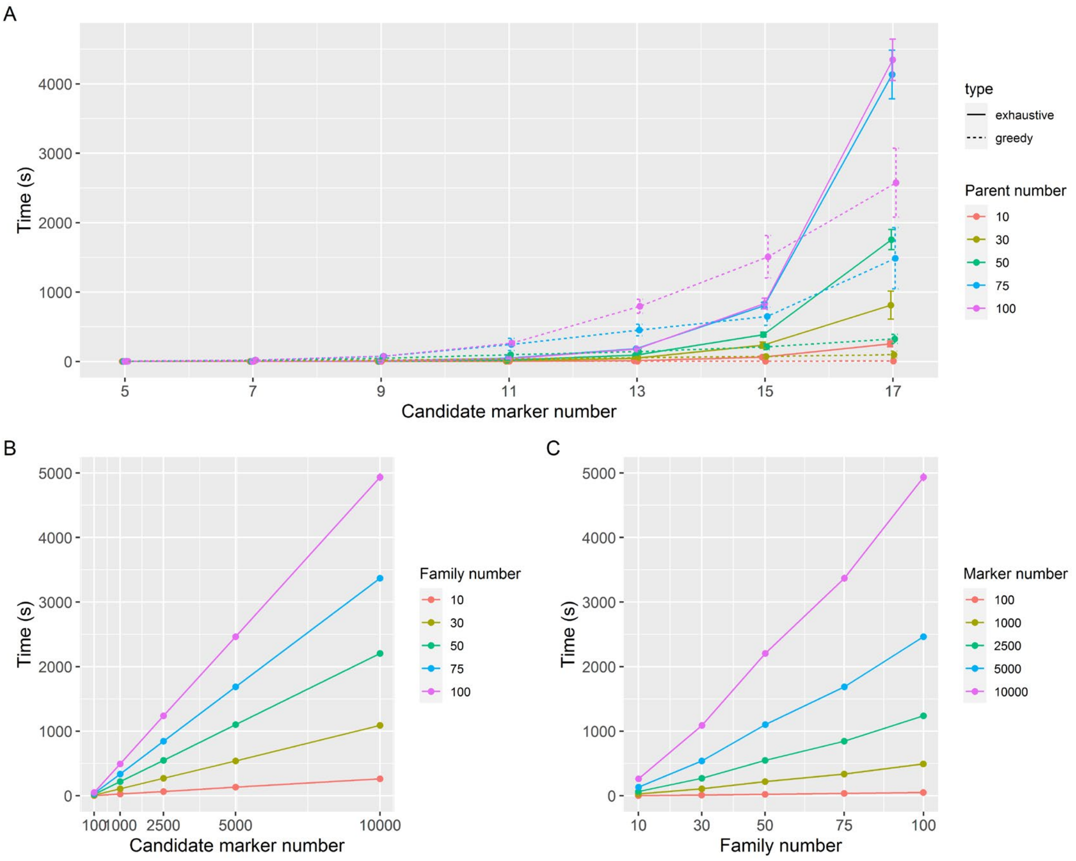

Figure S1: The running time of the exhaustive algorithm and the greedy algorithm under different number of candidate markers or parent pairs . A: The running time of the two algorithms when the number of candidate markers was small. B: The relationship between the marker number and the running time of the greedy algorithm for various family numbers. C: The relationship between the family number and the running time of the greedy algorithm for various marker numbers.

Section S2: Application in a real case

2.1 Methods

In order to investigate the factors which impact parentage assignment efficacy of the PMSs screened by the greedy algorithm (greedy-PMSs), we chose the genotyping data of a SSR marker set and 4 SNP marker sets from the Mexican gray wolf [19] as the real data sets (see the Results section for details of each data set). Since the greedy algorithm screens markers based on the genotypes of all candidate parents, we only used the eighteen trios within which both the parents and children were genotyped, and no missing markers for all parents. These trios belonged to 6 pedigrees. Considering that a large number of SNP markers were missing in MGW_1399, the only offspring of a certain trio, this trio was excluded in analysis based on SNP markers. Thus, 18 trios were used for SSR-based analysis, while 17 trios were used for SNP-based analysis. Parentage assignment was performed using CERVUS 3.0 (Kalinowski et al., 2007) and COLONY 2.0 (Jones & Wang, 2010) respectively based on the genotypes of PMS in corresponding offspring. In order to counteract the impact of erroneous or missing genotypes of markers in the PMS, we increased the redundancy of the PMS. To do so,

for SSR datasets, due to the high polymorphism, the first marker of a PMS was removed from the candidate marker set, and the second PMS was obtained, then the two PMS were merged to form the third PMS where the complement markers in the second PMS were served as redundant markers in case markers in the first one encountered genotype errors. For SNP datasets, since almost all markers were dimorphic, we excluded all markers in the first PMS for screening the second one where the markers in the second one were served as redundant markers.

As for the software used for parentage analysis, CERVUS 3.0, Critical Delta, the parameter which evaluates the reliability of parentage assignment results, is difficult to obtain significant results in case that the number of markers is small (the number of markers obtained by greedy algorithm is 3~5), and the software was designed for random mating populations instead of specified families, which conflicts with this case. Therefore, in this study, two criteria were used for the screening of parentage assignment results: ① It must be an authentic parent pair; ② TRIO LOD should be the largest and positive. As a matter of fact, incorrect or missing genotyping is not uncommon, and parentage assignment tended to fail for offspring with genotypable markers less than a half of the total markers in a non-redundant PMS. As for the another software, COLONY 2.0, the following settings were utilized: ① Monogamy for males and females was specified according to the authentic parent pairs; ② As a closed population, probabilities that the father and mother of an offspring were included in candidates were both set as 1; ③ No known sibship was specified; ④ The "Fulllikelihood" method was specified according to (Jones & Wang, 2010); ⑤ For each offspring, the authentic parent pair with the highest probability was ticked as the final result.

2.2 Results

We used one SSR dataset and four SNP datasets from a real case [19] to test the accuracy of parentage assignment through the greedy-PMSs. For each dataset, we screened two greedy-PMSs, merged them to form the third PMS, and used these three PMSs for parentage assignment (Table 4).

The two greedy-PMSs from the SSR markers included three markers (Locus 14, Locus 4, and Locus 16) and four markers (Locus 4, Locus 5, Locus 16, and Locus 18) respectively. When assigned using CERVUS, by only using the first greedy-PMS for parentage assignment, there were two samples failed to be traced back to their authentic parent pairs because of the incorrect genotypes on Locus 14 and Locus 4. Therefore, the parentage assignment accuracy of all 18 progenies was 88.89% (16/18). It is worth noting that Locus 4 was also present in the second greedy-PMS, but it did not lead to a failure because the other markers in the second greedy-PMS were sufficient for parentage assignment of it, so the accuracy of this PMS was 100%. With the second greedy-PMS served as redundant markers in case the first one encountered genotype errors, the accuracy of the third PMS also reached 100%. When it comes to COLONY, the accuracy turned 100% for all three PMSs, which implied a more accurate outcome of parentage assignment than CERVUS with SSR markers.

For the four SNP datasets, we compared the parentage assignment for 17 progenies. From the perspective of CERVUS, when one marker in a PMS was incorrectly genotyped in a sample, the parentage assignment for that sample failed, and the success rate was 0. In contrast, in most cases, the success rates were not affected until nearly half of the markers in a PMS were not genotyped (i.e., genotype missing). The only exception was PMS 45-1, whose markers showed highly similar genotypes in two parent pairs differed only at a missing locus. CERVUS preferentially outputted the parent pair that contains more homozygotes (Jones et al. 2010), and unluckily, the other one was correct. The similar results were obtained using COLONY, suggesting that as a single PMS, genotype error showed a more significant negative impact on parentage assignment. As for the low-redundancy PMS pooled from two greedy-PMSs, the accuracy increased for almost all datasets using both tools, suggesting that modestly increasing the redundancy of a PMS can effectively eliminate the negative impact of missing genotypes and genotype errors.

Section S3: Analysis of polymorphism and exclusion probability

3.1 Methods

The polymorphism information content (PIC, [22]) was adopted as the index to measure a single marker's polymorphism in a population, and it was calculated as follows:

$$PIC = 1 - \sum_{i=1}^{n} p_i^2 - (\sum_{i=1}^{n} p_i^2)^2 + \sum_{i=1}^{n} p_i^4$$

where p_i refers to the allele frequency of the marker's i-th genotype in the population. The higher the PIC, the higher the polymorphism of the marker in the population, which means a stronger efficacy for parentage assignment.

Since the parent genotypes were known in this study, the exclusion probability (PE) of a single marker could be calculated according to the following formula [7]:

$$PE = 1 + 4 * \sum p_i^4 - 4 * \sum p_i^5 - 3 * \sum p_i^6 - 8 * \left(\sum p_i^2\right)^2 + 8 * \sum p_i^2 * \sum p_i^3 + 2 * (\sum p_i^3)^2$$

where p_i refers to the allele frequency of the i-th genotype of the current marker. The greater the PE, the stronger the parentage assignment efficacy.

To evaluate the effectiveness of a set of markers for parentage assignment, it is necessary to comprehensively consider the PEs of all markers to form a cumulative exclusion probability (CPE). For a PMS, $CPE = 1 - \prod(1 - PE_i)$, where PE_i is the PE value of the i-th mark in the PMS.

Section S4: pseudo code for PMSeeker

Algorithm S1 Exhaustive Algorithm for PMS selection

Input: Parents' genotype file, block/family/group INFO

Output: Output file

function Main(ParentGeno, Info, Marker)

    Parameters: ParentGeno is the genotypes of all parents at all loci;

Info is the group information of all candidate parents, including either GENDER or FAMILY;

Marker is the list of currently tested markers

/*Output candidate Parent pairs*/
if Info == Gender **then** /*GENDER information is provided*/
    ParentPair ← all possible combinations between female and male
else /*Family is provided*/
    ParentPair ← one row one combination
end if
    /*Iteratively select a certain number of candidiate markers*/
for I in [1, len(Marker)] **do**
    for MS in all combinations of I markers **do**
        for pp in ParentPair **do**
            Gpp,MS ← $\{\boldsymbol{G_{i\times j,1}}, \boldsymbol{G_{i\times j,2}}, \boldsymbol{G_{i\times j,I}}\}$ /*offspring genotypes of pp (i×j) at marker set MS*/
        end for
        intersect ← False
        for pp_0 and pp_1 in ParentPair **do**
            if Gpp_0,MS ∩ Gpp_1,MS **then** /*pp_0 and pp_1 share the same offspring genotypes*/
                intersect ← True; break
            end if
        end for
        if intersect == False **then**
            output current marker set MS
        end if
    end for
end for
end function

Algorithm S2 Greedy Algorithm for PMS selection

Input: Parents' genotype file, block/family/group INFO

Output: Output file

```
function Main(ParentGeno, Info, Marker)
    Parameters: ParentGeno is the genotypes of all parents at all loci;
        Info is the group information of all candidate parents, including either GENDER or FAMILY;
        Marker is the list of currently tested markers
    if Info == Gender then                                  /*GENDER information is provided*/
        ParentPair ← all possible combinations between female and male
    else                                                    /*Family is provided*/
        ParentPair ← one row one combination
    end if
        root ← ParentPair                                   /*set all candidate parent pairs as the root*/
        N_l ← root; l = 1                                   /*initialize for the first layer 1*/
    while any |N_l| ! = 1 do
        /*select the LOCAL BEST marker for layer l*/
        for *m in Marker do
            for i in N_l do
                *S_{l,i} ← {P_{a1}P_{b1}, P_{a2}P_{b2}, ...}        /**Nl,i contains {P_{a1}P_{b1}, P_{a2}P_{b2}, ...}*/
                *G_{l,i} ← {G_{a1×b2} ∪ G_{a2×b2} ∪ ... }
                for *G_{l,i,x} in *G_{l,i} do
                    *G_{l,i,x} ← PPs in *S_{l,i} that generate G_{l,i,x}
                end for
            end for
            *N_{l+1} ← {*G_{l,1},* G_{l,2}, ...}
            deduplicate *N_{l+1} and tick out *N_{l+1,i} == 1
            calculate PDI for *N_{l+1}
        end for
        Choose the MARKER with the minimum PDI as the LOCAL BEST marker
        /*output the N_{l+1}*/
        N_{l+1} ← LOCAL BEST marker's *N_{l+1}
```

```
        l ← l + 1
    end while
end function
```
